# Dental erosion in patients seeking treatment for gastrointestinal complaints: a case series

**DOI:** 10.1186/s13256-015-0738-x

**Published:** 2015-10-30

**Authors:** Vincenzo Bruno, Massimo Amato, Santo Catapano, Paola Iovino

**Affiliations:** 1 Dental School, University of Ferrara, Via Savonarola 9, 44100 Ferrara, Italy; 2 Department of Medicine and Surgery, University of Salerno, Via S Allende, 84081 Baronissi, Italy

**Keywords:** Dental erosion, Diagnosis, Eating disorders, Functional dyspepsia, Management, Rome III criteria

## Abstract

**Introduction:**

Eating disorders which embrace anorexia nervosa, bulimia nervosa, and eating disorders not otherwise specified can be life-threatening due to general medical complications; however, the diagnosis of eating disorder is often delayed due to a low suspicion index. Gastroenterologists are health care providers who may come into contact with patients with undiagnosed eating disorders; it has been previously demonstrated that patients with eating disorders frequently have a significant association with functional dyspepsia. Signs of dental erosion have been described in patients with eating disorders; hence, they may help to identify eating disorders in patients who present with functional dyspepsia and deny having an eating disorder.

**Case presentation:**

In this report we describe three cases (a 25-year-old white woman, a 24-year-old white woman, and a 40-year-old white man) with undiagnosed eating disorders, in which a more comprehensive approach, such as the recognition of dental erosion joined with a careful gastrointestinal investigation, was performed to reach a final diagnosis of an eating disorder.

**Conclusions:**

The screening for dental erosion in patients seeking or receiving medical treatment for dyspeptic symptoms in a gastrointestinal out-patient clinic could be an aid for gastroenterologists to recognize the presence of an underlying eating disorder.

A close collaboration with dentists, in addition to psychiatrists, could provide a more favorable treatment outcome.

## Introduction

Eating disorders (EDs) are characterized by a persistent disturbance of eating that impairs health or psychosocial functioning. Three main ED categories have been classified by the American Psychiatric Association’s *Diagnostic and Statistical Manual of Mental Disorders*, Fifth Edition (*DSM*-5) [1]: anorexia nervosa (AN), bulimia nervosa (BN), and eating disorders not otherwise specified (EDNOS). In a recent nationally representative US survey, the estimated lifetime prevalence of AN and BN was 0.6 % and 1.0 % respectively and it was consistently 1.75 to 3 times higher among women as men [2]. EDs are associated with the highest rates of morbidity and even mortality of any mental disorders especially among adolescents. Failure to recognize their early signs can compromise a patient’s recovery and long-term prognosis.

A large number of individuals seeking or receiving medical treatment for gastrointestinal (GI) symptoms in GI out-patient clinics could mask a concurrent often undiagnosed ED [3, 4]. However, the early recognition of an ED is sometimes very difficult.

It has been recently demonstrated that patients with ED, when carefully investigated, significantly showed a high prevalence of dyspeptic symptoms fulfilling Rome III criteria for functional dyspepsia (FD). FD is one of the most common functional GI disorders (FGID) [4]. In particular, patients with FD complain of frequent meal-related symptoms and are diagnosed as having post-prandial distress syndrome (PPDS), which is a subtype of FD. Tooth erosion has been reported as an oral manifestation that might help in the early detection of EDs [5]. Hence, gastroenterologists should be encouraged to routinely screen for EDs in patients who present with FD/PPDS and deny having an ED.

In this report we describe three cases with undiagnosed ED that came under our observation in a GI out-patient clinic, in which a more comprehensive approach paved the way for reaching a final diagnosis and, eventually, successful treatment.

## Case presentation

### Case 1

**Fig. 1:**
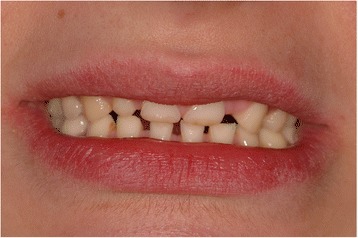
Severe alteration of the patient’s smile

A 25-year-old white woman was referred to the Gastrointestinal Unit of the University of Salerno for severe constipation. After having already followed a long treatment with Movicol (macrogol; Norgine Ltd, Hengoed, UK) with no resolution to her constipation, she underwent a rigorous assessment process to exclude any organic diseases, including proctosigmoidoscopy as well as laboratory tests. No abnormalities were found and she fulfilled Rome III criteria for functional constipation. Further diagnostic tools were then planned to better classify her functional constipation including a colonic transit time and an anorectal manometry with a balloon expulsion test, which indeed showed slow transit time constipation. During the routine physical examination, an abnormality of her teeth was perceived and a dental evaluation was requested (Fig. 1).

**Fig. 2:**
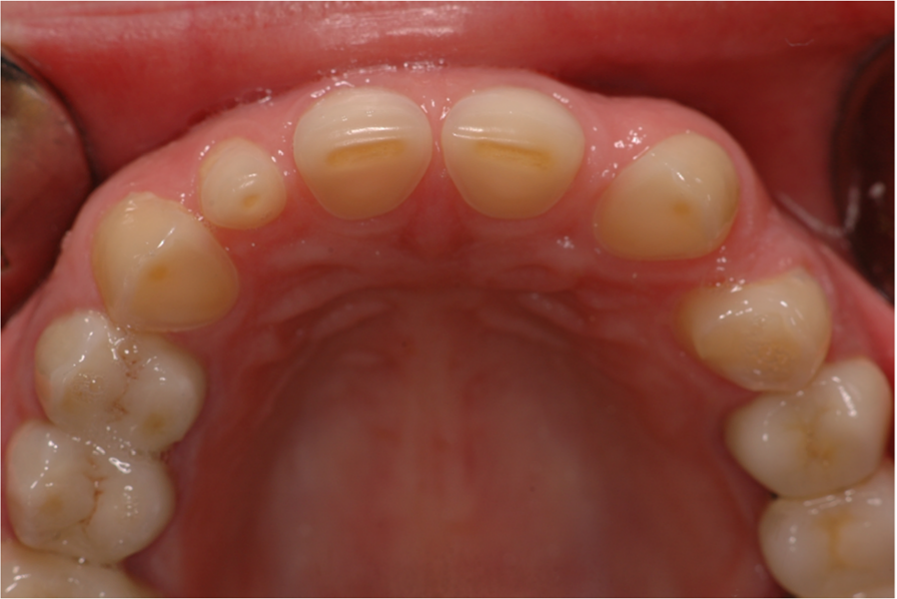
Erosion with the involvement of dentin

The dentist diagnosed dental erosion of the maxillary anterior teeth, in particular the lingual and incisal surfaces (Fig. 2). Then, he asked the patient about any previous episodes of recurrent vomiting, of any eating habits such as Coke-swishing or vegetable and/or fruit-mulling; however, her answer was negative. In consequence, the dentist had a meeting with gastroenterologists and the suspicion of an ED was raised. At the second more careful gastroenterological assessment, the patient also tested positive for FD, specifically PPDS. Therefore, the suspicion of an ED became even stronger, and a psychiatric evaluation was planned reaching the final diagnosis of AN, which required psychiatric treatment.

### Case 2

**Fig. 3:**
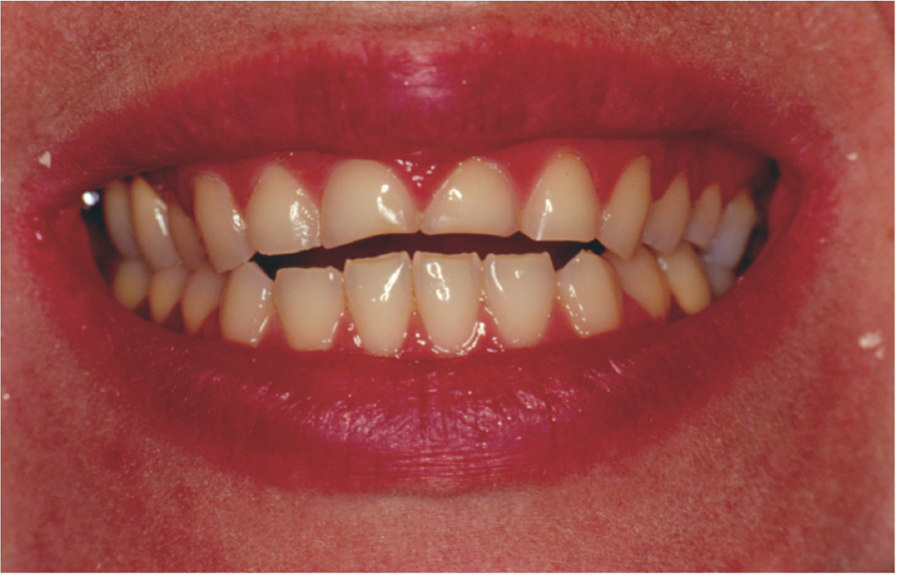
The altered smile-line caused by the erosion of the incisal edges

The second case study was a 24-year-old white woman who visited our dental clinic. Her main complaint was esthetic (Fig. 3); she wished to reestablish her smile. A clinical examination showed that her maxillary anterior teeth and her first two maxillary premolars presented erosion on the lingual and incisal surfaces.

**Fig. 4:**
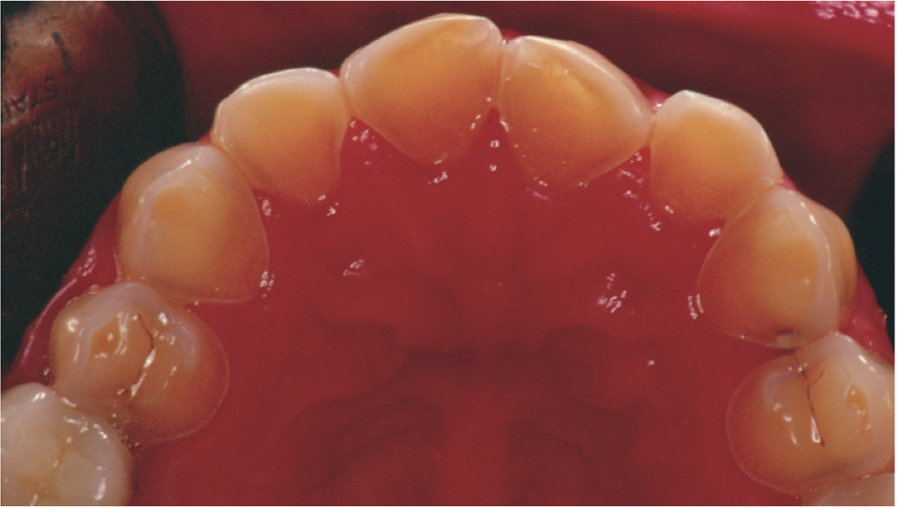
Intra-oral view with the typical appearance of dental erosion

A complete medical assessment was obtained, and no relevant medical risk was reported. However, during the intra-oral assessment the aspect of the dental erosion suggested recurrent vomiting (Fig. 4), but she denied vomiting and having eating habits such as Coke-swishing or vegetable and/or fruit-mulling. Since she also had a body mass index (BMI; weight in kilograms divided by height in meters squared) of 18.5, she was referred to our GI out-patient clinic to investigate this unexplained weight loss. In our tertiary level-of-care referral center she underwent a careful medical history with detailed dietary information and physical examination. She had been complaining of a vague sensation of fullness while eating for years, especially to lipids, but continued to deny any vomiting. In the absence of any other alarm symptoms such as fever, GI bleeding, and abdominal mass, routine laboratory tests were performed that showed a mild anemia (hemoglobin; Hb 10.1 mg/dl) with low serum ferritin (8 μg/mL). She was then tested for the presence of anti-transglutaminase antibodies and anti-endomysium antibodies to rule out celiac disease. The results were negative after excluding immunoglobulin A (IgA) deficiency. The breath test for *Helicobacter pylori* was negative. She fulfilled the Rome III criteria for PPDS. The PPDS diagnosis and the dental erosion led us to suspect an ED and a psychiatric evaluation was requested. To avoid the possibility of losing the patient because her first wish was to reestablish her smile, a combined therapeutic option was suggested, which involved psychiatric, gastroenterological and dental treatment. She accepted. However, she failed to attend the follow-up appointment at our GI out-patient clinic.

### Case 3

**Fig. 5:**
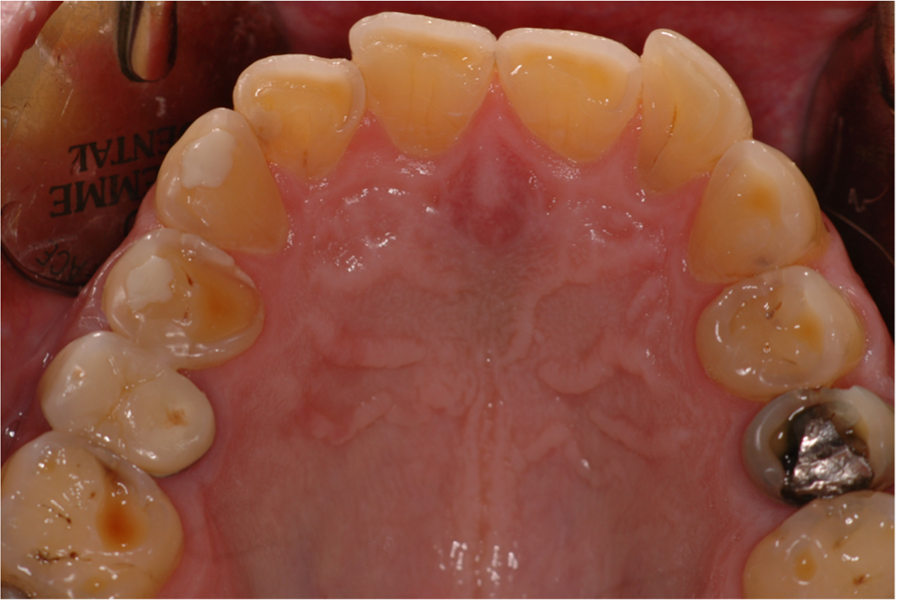
Erosion of the palatal surfaces and intact enamel borders along the gingival margin

The third case was a 40-year-old white man who came into our GI out-patient clinic complaining of regurgitation. A careful medical history was obtained and dyspeptic symptoms were investigated and accurately distinguished from symptoms more suggestive of esophageal, pancreatic, or biliary disease. Neither prescription nor nonprescription medications commonly associated with dyspepsia, especially non-steroidal anti-inflammatory drugs (NSAIDs), nor eating habits such as Coke-swishing or vegetable and/or fruit-mulling were identified. No alarm symptoms such as GI bleeding, unexplained weight loss or fever were disclosed and laboratory evaluations were negative, so FD was diagnosed. During a routine physical examination alterations to his teeth, similar to those that were previously described in Case 1, were observed and a screening for dental erosion resulted positive. Prior to any pharmacological treatment, he was referred to our dental clinic. The dentist ruled out that the main complaint was dentine hypersensitivity and confirmed, at oral examination, mild erosions on the maxillary anterior teeth (1.4 to 2.4). These erosions were present only at the lingual surface (Fig. 5) leading to a strong suspicion of ED. In this case, the patient was referred to a psychiatric clinic and he accepted. Finally, the diagnosis of a bulimic disorder according to *DSM*-5 was made and he started psychiatric treatment together with prokinetic agents to address PPDS complaints and dental treatment. He is now at follow-up and satisfied with our management.

## Discussion

Gastroenterologists are health care providers who may come into contact with patients with an undiagnosed ED early on. In fact, it is a common occurrence that patients, before presenting to health care services with an ED, seek treatment for GI symptoms [4], such as dyspeptic symptoms that induce high utilization of health care and have a negative impact on quality of life [6]. Dyspeptic symptoms are very common with a prevalence of approximately 20 to 25 % in Western countries; the prevalence is slightly higher in women [7].

Currently, internationally accepted clinical criteria (Rome III criteria) are extensively used to diagnose FGIDs [6]. The Rome III Criteria were developed by a committee that recommended the pragmatic description of FD to be the presence of symptoms such as epigastric pain, epigastric burning, post-prandial fullness and early satiation in the absence of any organic, systemic, or metabolic disease that is likely to explain the symptoms. Furthermore, Rome III criteria help to distinguish whether patients report symptom aggravation after ingestion of a meal, the so-called PPDS characterized by postprandial fullness and early satiation, or meal-unrelated dyspeptic symptoms, the so-called epigastric pain syndrome (EPS), characterized by epigastric pain and epigastric burning [6]. A distinction between meal-related and meal-unrelated symptoms has been demonstrated as clinically relevant for disclosing differences across EDs [4]. In our case report, the presence of dental erosions together with the presence of dyspeptic symptoms fulfilling the diagnosis of FD on the basis of Rome III criteria led to the diagnosis of ED. It is well known, however, that patients with an ED commonly refuse to admit having a problem [8, 9]. For example, our first patient denied any vomiting during the assessment at the dental clinic and only the presence of typical erosion raised the suspicion of an ED.

Dental erosion is commonly described as a progressive loss of hard dental tissue that is unrelated to bacterial activity. Erosion needs to be differentiated from attrition, normal physiological wear of teeth, and abrasion which is the pathologic wear of teeth caused by an involuntary action that is teeth grinding [5, 10].

The major cause of dental erosion is a chemical action associated with habitual vomiting or regurgitation or the eating of acid foods producing progressive wear of the anterior teeth in the maxillary arch. This is caused by the direction of the vomit as well as the position of the tongue during the episodes. The lingual surfaces of the maxillary anterior teeth are disintegrated by the acid during vomiting episodes, including the palatal part of the maxillary posterior teeth. In contrast, the mandibular anterior teeth appear normal since the tongue protects them. Cratering or cupping could be present, and amalgam restorations, if present, appear raised. It has been previously demonstrated that it could be important to differentiate dental erosion caused by habitual vomiting or regurgitation from that caused by eating habits such as Coke-swishing or vegetable and/or fruit-mulling [10–12]. The latter can be easily excluded by taking a careful medical record because patients usually admit to eating these kinds of food and beverages and have the typical appearance of wear abnormalities. In a recent systematic review and meta-analysis, purging practices seem to increase the risk of tooth erosion. Nevertheless, there is a significant lack of scientific evidence to fulfill the basic criteria of causation between tooth erosions and EDs [5]. Other oral signs such as sialadenosis and oropharyngeal erythema have been previously described more frequently in patients with anorexia and bulimia and could be taken into account to suspect an ED. In our three cases, dental erosion captured our attention; gastroenterologists would concentrate on the presence of sialadenosis [13] but it is quite difficult to perform this assessment.

Our combined (gastroenterologists and dentists) approach to the patients raised the suspicion of an ED and although communication with patients with an ED is very difficult, our approach helped patients to admit their ED problem and accept the psychiatric help that is the first step to ensuring future optimal treatment success.

The hallmarks of EDs are clinical disturbances in body image and eating behavior resulting in physical and psychological impairment. AN, BN and EDNOS are more common in women and can result in long-term health consequences even in increased mortality. The core presentation of AN is characterized by the inability or refusal to maintain a minimally normal weight, and a profoundly distorted perception of body weight and shape. Amenorrhea commonly occurs in AN. Under the definition of BN are included individuals with comorbid psychopathology who engage in recurrent binge-eating episodes and recurrent inappropriate compensatory behaviors that are intended to rid calories that they voraciously ingested. EDNOS involves milder versions of AN and BN that do not satisfy all the criteria [1]. Previous studies have suggested that patients with anorexia frequently complain of GI symptoms hinting at a disordered gastric motility, especially when they are in a refeeding phase [14]. Dyspeptic symptoms such as epigastric fullness and distension were found to be significantly more prevalent and intense than in healthy individuals and may serve as an argument for food refusal [15]; however, they are often overlooked or misinterpreted. In patients with bulimia the large quantities eaten during a binge not only lead to a feeling of loss of control but also to a sensation of epigastric distension. The latter as well as the often associated epigastric pain are terminated by self-induced vomiting, which allows either continuation or termination of the binge. The mechanisms underlying dyspeptic symptoms in EDs are still unclear, although malnutrition and the resultant metabolic myopathy, along with electrolyte depletion seem to play a crucial role in determining the demonstrated abnormalities in gastric emptying, gastric capacity and blunted endocrine control [14].

Once established, the psychological and physiological GI disturbances can perpetuate and strengthen each other resulting in an FGID that can persist independently of the ED that originally caused the motor and sensitivity disturbances [16].

A limitation of this study is the failure to register the presence of sialadenosis [13].

## Conclusions

It is conceivable that a large number of individuals receiving medical treatment for meal-related symptoms in GI out-patient clinics could be better managed by the identification of a concurrent ED. This is an important issue given that the ultimate goal of therapy in patients with a suspected ED is the normalization of gastric motor function with the resumption of normal eating behavior enabling the patient’s social reintegration and restoration to an appearance acceptable to the social environment.

However, these cases highlight the difficulties in reaching a diagnosis of ED and should encourage the routine screening for oral signs of recurrent vomiting in patients fulfilling the Rome III Criteria for PPDS in GI out-patient clinics.

Special attention should be given to detect subclinical cases of EDs to avoid the progression of disease and further comorbidities that expand its clinical impact across different specialties.

## Consent

Written informed consent was obtained from the patients for publication of these case reports and accompanying images. A copy of the written consents is available for review by the Editor-in-Chief of this journal.
